# Gut Microbiota and Neurotransmitter Regulation: Functional Effects of Four Traditional Chinese Fermented Soybean (Sojae Semen Praeparatum)

**DOI:** 10.3390/foods14040671

**Published:** 2025-02-16

**Authors:** Lin Zhang, Huo Su, Siqi Wang, Yujie Fu, Manyuan Wang

**Affiliations:** Beijing Key Laboratory of TCM Collateral Disease Theory Research, School of Traditional Chinese Medicine, Capital Medical University, Beijing 100069, China

**Keywords:** fermentation processes, functional food, intestinal homeostasis, neurotransmitter regulation

## Abstract

This study aims to evaluate the potential disease prevention and treatment functions of four types of traditional Chinese fermented Sojae Semen Praeparatum (SSP) by analyzing their nutritional active components and their effects on the gut microbiota. Raw soybeans and the four SSPs were administered as dietary supplements to normal SD rats for 6 weeks. Fecal samples were collected at weeks 0, 2, and 6 to assess changes in the gut microbiota. Our results revealed that different fermentation methods resulted in variations in soybean isoflavone content. Fermented soybeans promoted the growth of beneficial microorganisms associated with short-chain fatty acid production in the gut microbiota, such as *Christensenellaceae_R_7_group*, compared to unfermented soybeans. Supplementation with SSPs fermented with different processes increased the diversity of the rat gut microbiota, except for the fermented group of qingwenjiedu decoction (QW). The dominant gut microbiota in the fermented group of Artemisia Annuae Herba and Mori Folium (QS) exhibited anti-inflammatory effects, while the dominant gut microbiota in the fermented group of Ephedrae Herba and Perillae Folium (MZ) showed antidepressant effects. In the neurotransmitter analysis, MZ reduced gamma-aminobutyric acid (GABA) levels, the fermented group without Chinese medicine (DD) decreased dopamine levels, and both QS and QW increased norepinephrine levels. Correlation analysis highlighted connections between gut microbiota, neurotransmitters, and chemical levels. The results indicate that SSPs may contribute uniquely to health by maintaining intestinal balance and improving neurological disorders while predicting a potential association between neurotransmitters and gut microbiota by correlation analysis.

## 1. Introduction

Fermentation is a traditional and globally significant method for food preservation and processing. In Asia, traditional fermentation processes yield various products, including tempeh from Indonesia, natto from Japan, doenjang from Korea, and douchi from China [1]. Recently, fermented foods have received widespread attention due to their distinct organoleptic properties and emphasis on health maintenance [2]. Fermented foods contain three main functional components in varying amounts: functional microorganisms (probiotics), prebiotics, and biogenic metabolites (such as polyphenols and amino acids) that make fermented foods functionally active [3]. The functional components present in fermented foods play crucial biological roles in the gastrointestinal tract and could influence the composition of the gut microbiota upon consumption. Emerging evidence highlights the therapeutic potential of fermented foods in mitigating dysbiosis-associated pathologies, such as inflammatory bowel disease (IBD), atherosclerosis, and neuroinflammatory disorders, via microbiota-mediated modulation of host immune–metabolic pathways [4,5,6].

Fermented soybeans have been preferred by Asian consumers as a functional food over the years. Fermenting soybeans enhances the bioavailability of flavonoids and proteins and improves their flavor and stability [7]. It promotes human health by regulating intestinal stability, enhancing gut barrier function, modulating gut microbiota, and producing short-chain fatty acids (SCFAs) [8,9,10,11]. Contemporary medical experiments have indicated an elevation in the gamma-aminobutyric acid (GABA) content of fermented soybeans [12]. Notably, GABA-enriched fermented beans have demonstrated effective relief of mild depression-like symptoms in a mouse model [13]. Additionally, the fermentation process breaks down isoflavone glycosides, releasing isoflavone aglycones, including genistein, daidzein, and glycitein [14,15]. These isoflavones inhibit acetylcholinesterase (AChE) activity, increasing acetylcholine (ACh) levels and modulating the cholinergic system and neuronal function [16]. These findings have prompted researchers to investigate the potential effects and mechanisms of fermented soybeans on the human gut system.

Fermented soybeans are available in diverse forms, including douchi, tofu, natto, and soy sauce, especially in East Asian countries and regions. Douchi, a distinctive traditional Chinese fermented soybean product, is primarily prepared through the natural fermentation of soybeans, combined with salt, liquor, pepper, Chinese herbal medicine, and assorted spices. In traditional literature, certain types of douchi were utilized as edible Chinese herbal medicines, such as dandouchi (Sojae Semen Praeparatum) (SSP). SSP is used as a Traditional Chinese Medicine (TCM) to relieve external vexation by dispelling stagnation and heat and is often used for chest tightness, irritability, and insomnia [17]. The existing conventional methodology for the production of SSP is various, primarily adhering to the guidelines established by the Chinese Pharmacopoeia and regional processing standards. SSP can be made from soybeans by pure breed and natural fermentation. SSP was observed to enhance and modulate hypothalamic–pituitary–adrenal axis (HPA) hyperactivity and gut microbiota composition [18]. Furthermore, SSP fermented from pure strains exhibited the capability to improve gut microbiota, restore neurotransmitter levels, and alleviate depressive behavior in rats [9]. However, natural fermentation processes for the preparation of SSP are varied, as documented in the Chinese Pharmacopoeia and local processing standards [19]. Concerning studies on SSPs using different fermentation processes, only a few reports have demonstrated noteworthy differences in chemical composition among the varieties [20,21]. Limited studies have concentrated on the alterations in gut microbiota following the intervention of naturally fermented SSP in disease model animals. Meanwhile, comprehensive investigations into the interactions between gut microbiota and neurotransmitters in normal rats through SSP have been largely lacking.

Based on the current study, we have selected four processes under the Pharmacopoeia of the People’s Republic of China and local processing standards. The aims of this research are (i) to evaluate the regulation of gut microbiota in normal rats by consuming natural fermented SSPs for six weeks and (ii) to investigate the similarities and differences in the modulation of gut microbiota and neurotransmitters resulting from the consumption of various processes of SSPs in normal rats. This research aims to elucidate the influence by which various types of SSPs positively influence gut microbiota and neurotransmitter levels. Furthermore, it seeks to advance the research, development, and application of functional health foods derived from SSPs that have undergone diverse fermentation processes, thereby enhancing overall human health.

## 2. Materials and Methods

### 2.1. Material and Reagents

Black soybean (*Glycine max* (L.) Merr), Artemisiae Annuae Herba (*Artemisia annua* L.), and Mori Folium (*Morus alba* L.) were purchased from Anguo (Baoding, China). Ephedrae Herba (*Ephedra sinica* Stapf), Perillae Folium (*Perilla frutescens* (L.) Britt.), Qingwenjiedu decoction: Angelicae Dahuricae Radix (*Angelica dahurica* (Fisch. ex Hoffm.) Benth. et Hook. f.), Scrophulariae Radix (*Scrophularia ningpoensis* Hemsl.), Bupleuri Radix (*Bupleurum chinense* DC.), Forsythiae Fructus (*Forsythia suspensa* (Thunb.) Vahl), Platycodonis Radix (*Platycodon grandiflorum* (Jacq.) A.DC.), Chuanxiong Rhizoma (*Ligusticum chuanxiong* Hort.), Scutellariae Radix (*Scutellaria baicalensis* Georgi), Notopterygii Rhizoma et Radix (*Notopterygium incisum* Ting ex H. T. Chang), Paeoniae Radix Rubra (*Paeonia lactiflora* Pall.), Puerariae Lobatae Radix (*Pueraria lobata* (Willd.) Ohwi), Lophatheri Herba (*Lophatherum gracile* Brongn.), Glycyrrhizae Radix et Rhizoma (*Glycyrrhiza uralensis* Fisch.), and Zingiberis Rhizoma Recens (*Zingiber officinale* Rosc.) were purchased from the Pharmacy of Beijing Hospital of Traditional Chinese Medicine (Beijing, China).

Genistein (111709-200501, 99.1%), genitin (111709-200501, 99.0%), daidzein (111502-200402, 99.0%), and daidzin (111738-201302, 91.3%) were obtained from the China National Institute for Food and Drug Control (Beijing, China). Glycitein (LE40Q149, 98%) was obtained from Beijing Bailingwei Technology Co., Ltd. (Beijing, China). Aspartic acid, glutamic acid, serine, glycine, threonine, proline, alanine, gamma-aminobutyric acid (GABA), tryptophan, methionine, valine, phenylalanine, isoleucine, leucine, lysine, histidine, and arginine (purity level ≥98%) were obtained from Shanghai yuanye Bio-Technology Co., Ltd. (Shanghai, China). O-phthalaldehyde (OPA) and β-mercaptoethanol (MCE) were purchased from Sigma (St. Louis, MO, USA). Sodium tetraborate decahydrate (Na_2_B_4_O_7_⋅10H_2_O) was purchased from Merck (Darmstadt, Germany). Acetonitrile and methanol (HPLC grade) were provided by Fisher (Fair Lawn, NJ, USA). Methanol and phosphoric acid (analytical grade) were obtained from Beijing Chemical Works (Beijing, China).

Enzyme immune assay kits for determining gamma-aminobutyric acid (GABA), noradrenaline (NE), dopamine (DA), and acetylcholine (ACH) levels were obtained from Nanjing Jian Cheng Biological Technology Co., Ltd. (Nanjing, China).

### 2.2. The Fermenting Processes of SSP

**Figure 1:**
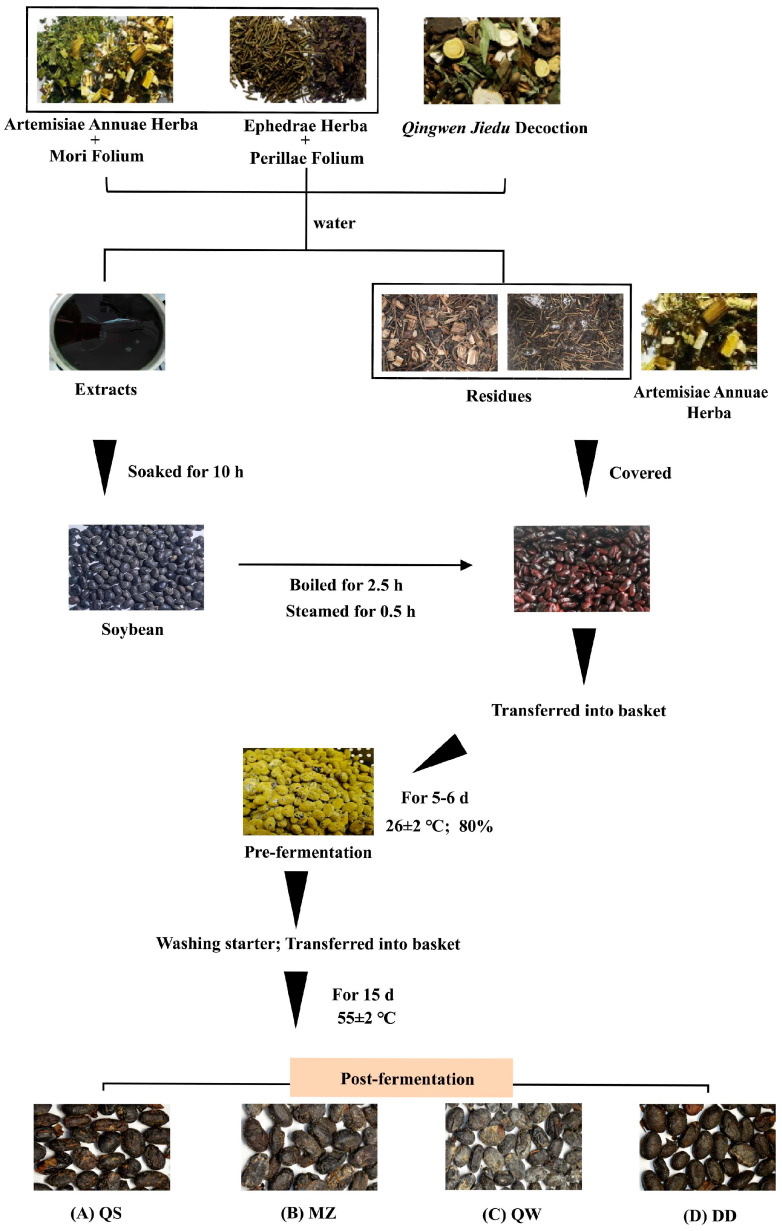
Flowchart of traditional methods of preparation of SSPs. (**A**) QS, (**B**) MZ, (**C**) QW, (**D**) DD.

In this study, we use the Pharmacopoeia of the People’s Republic of China and local processing standards, along with traditional Chinese medical books such as *Medical Complete Book*, *Ancient and Modern* and *The Compendium of Materia Medica*. We chose four representative preparations for laboratory-made SSP, incorporating various medicinal excipients during the fermentation process to obtain SSPs with distinct properties (Figure 1). The soybeans were washed, soaked with water/Chinese medicine extract for 10 h, and steamed for 2.5 h. The cooled soybeans were covered with Chinese medicine residue (QS, MZ)/Artemisiae Annuae Herba (QW) and placed in fermentation baskets. The mixture was subjected to incubation in a constant temperature and humidity chamber (HWS-150B, Tianjin Taisite Instrument Co., Ltd., Tianjin, China) at a temperature of 26 ± 2 °C for a duration of 5 to 6 days, maintaining a humidity level of 80%. This process continued until the emergence of abundant yellow or white hyphae, indicative of successful pre-fermentation, was observed. The mature starter was washed and sealed directly in a fermentation bag for anaerobic fermentation. The fermentation temperature and time were 55 ± 2 °C and 15 d, respectively. After being steamed for 0.5 h, the SSP was obtained and dried in a drying oven (GZX-9070MBE, Kunshan Ultrasonic Instrument Co., Ltd., Kunshan, China) at 60 °C. The appearance of the 4 types of SSPs varied in color under different processes. Among them, the DD group exhibited a strong odor, and the section appeared brown. The QS group had a distinct odor, and the section was yellowish-brown. The MZ group featured a clear odor, and the section appeared brownish-yellow. The QW group emitted a strong odor, and the section was brownish-yellow.

### 2.3. Extract and Analysis of SSPs

SSPs (4 types of SSP) and raw soybean were pulverized in a high-speed universal pulverizer (Tianjin Taisite Instrument Co., Ltd., Tianjin, China) using a 40-mesh screen (diameter of 0.425 mm, Shanghai Xunchuan Screen Mesh Co., Ltd., Shanghai, China) and stored under dry conditions at room temperature before analysis. SSPs and raw soybean powder were extracted twice with 80% ethanol under reflux (70 °C) for 1 h. The combined filtrates were concentrated under vacuum at 40–45 °C using a rotary evaporator (R-210, Büchi Labortechnik AG, Flawil, Switzerland) and then freeze-dried in a freeze dryer (FDL-2000, EYELA, Tokyo Rikakikai Co., Ltd., Tokyo, Japan) at −80 °C. The resulting samples were diluted with 75% ethanol and filtered through a 0.22 μm syringe filter. The contents of genistein, daidzein, and glycitein in the SSP and raw soybean extracts were analyzed using a Shimadzu LC-20A infinity (Shimadzu Corporation, Kyoto, Japan) with an Agilent ZORBAX Eclipse XDB-C18 (4.6 mm × 250 mm, 5 μm) column. Eluent A was 0.1% glacial acetic acid, eluent B was acetonitrile, and the elution was performed as follows: 0–5 min for B from 15–15%, 5–12 min for B from 15–20%, 12–32 min for B from 20–50%, 32–33 min for B from 50–85%, 33–40 min for B from 85–85%, and 40–41 min for B from 85–15% at a column temperature of 34 °C and a flow rate of 1 mL/min. The injection volume was 20 μL, and the detection wavelength was set at 260 nm.

The freeze-dried samples were extracted twice with petroleum ether by ultrasonication (KQ-800E, Kunshan Ultrasonic Instrument Co., Ltd., Kunshan, China). The extract was centrifuged at 5000 rpm, the supernatant removed and the residue retained. The residual petroleum ether was evaporated in a water bath at 60 °C in a fume cupboard to obtain the defatted sample powder. The defatted powder was extracted with pure water for 1 h. The supernatant was passed through a 0.22 μm microporous filter membrane, and the filtrate was the test solution. The contents of amino acid in the SSP and soybean extracts were analyzed using an Agilent 1260 Infinity LC System (G7121B, Agilent Technologies, Inc., Santa Clara, CA, USA) (1260II, Agilent Technologies, Inc., USA) with an Agilent ZORBAX SB-C8 column (2.1 mm × 100 mm, 3.5 μm) column. Eluent A was 60 mmol/L citrate buffer salt (pH = 6.0), and B was methanol; gradient elution (0~18 min, 21%B; 18~19 min, 21~28%B; 19~26 min, 28%B; 26~27 min, 28~32%B; 27~40 min, 32%B; 40~41 min, 32~36%B; 41~43 min, 36%B; 43~44 min, 36~41%B; 44~46 min, 41%B; 46~47 min, 41~46.5%B; 47~52 min, 46.5%B; 52~53 min, 46.5~49.5%B; 53~58 min, 49.5%B; 58~59 min, 49.5~59%B; 59~64 min, 59%B; 64~64.5 min, 59~70%B; 64.5~70 min, 70%B); flow: 1.0 mL/min; column temperature: 30 °C; injection volume: 2 μL; detection wavelength: excitation wavelength 338 nm, absorption wavelength 425 nm. Derivatization was performed online using an autosampler, and the derivatization procedure was as follows: take up 2.00 μL of sample; take up 8.00 μL of o-formaldehyde derivatizing agent; take up 0.50 μL of air; mix with air at maximum speed for 8 times and wait for 1 min; the sample after derivatization was injected into the liquid chromatograph.

### 2.4. Animals

Six-week-old male Sprague Dawley rats were purchased from Beijing Vital River Laboratory Animal Technology Co., Ltd. (Beijing, China) and were housed in a room maintained at 23 ± 3 °C with 50% ± 10% relative humidity and a 12-h light/dark cycle. They were given free access to food and water. After a week of adjustable feeding, the rats were randomly divided into six groups: control (NS; Saline), raw soybeans (SS), the fermented group without Chinese medicine (DD), the fermented group of Artemisia Annuae Herba and Mori Folium (QS), the fermented group of Ephedrae Herba and Perillae Folium (MZ), and the fermented group of qingwenjiedu decoction (QW) (*n* = 6 in each group). Daily food consumption and body weight were recorded. All experimental protocols were approved by the Institutional Animal Ethics Committee of Capital Medical University (AEEI-2016-046). Dosages were calculated based on the equivalent conversion of body surface area between animals and humans. As stipulated by the Pharmacopoeia of the People’s Republic of China, the recommended clinical dosage of SSP is between 6 and 12 g per day. To translate this dosage for rats following body surface area, the following calculation is applicable:

$$Animal\;dose\;(mg/kg)=Human\;dose\;(mg/kg)\times \frac{Animal\;BSA\;(m^{2})}{Human\;BSA\;(m^{2})}$$

Considering the established conversion factor for rats is 6.3, the minimum dosage of 6 g/day was multiplied by this factor, resulting in an administration of 37.8 g/kg to the rat subjects. Feces were collected from weeks 0, 2, and 6 of the intervention periods and stored at −80 °C. After six weeks of consumption, the rats were sacrificed under isoflurane anesthesia following a 12-h fast. Serum and colonic content were collected and stored at −80 °C until analysis.

### 2.5. Analysis of Neurotransmitter in Serum

Serum samples were centrifuged (CHT210R, Hunan Xiangyi Laboratory Instrument Development Co., Ltd., Changsha, China) for 10 min at 4 °C at 4000× *g*, and serum was stored in aliquots at −80 °C until assayed. The amounts of GABA, NE, DA, and ACH in the serum were measured using commercially available ELISA kits according to the manufacturer’s instructions and by analyzing with a multi-mode microplate reader (SpectraMax@ iD3, Molecular Device, San Jose, CA, USA).

### 2.6. Fecal Bacteria DNA Extraction and 16S rRNA Gene Sequencing

Microbial DNA was extracted from feces samples using the E.Z.N.A.^®^ soil DNA Kit (Omega Bio-tek, Norcross, GA, USA) according to the manufacturer’s protocols. The final DNA concentration and purification were determined by NanoDrop 2000 UV-vis spectrophotometer (Thermo Scientific, Wilmington, NC, USA), and DNA quality was checked by 1% agarose gel electrophoresis. The V3-V4 hypervariable regions of the bacteria 16S rRNA gene were amplified with primers 338F (5′-ACTCCTACGGGAGGCAGCAG-3′) and 806R (5′-GGACTACHVGGGTWTCTAAT-3′) by thermocycler PCR system (GeneAmp 9700, ABI, Foster City, CA, USA). The PCR reactions were conducted using the following program: 3 min of denaturation at 95 °C, 27 cycles of 30 s at 95 °C, 30 s for annealing at 55 °C, and 45 s for elongation at 72 °C, and a final extension at 72 °C for 10 min. PCR reactions were performed in triplicate 20 μL mixture containing 4 μL of 5 × FastPfu Buffer, 2 μL of 2.5 mM dNTPs, 0.8 μL of each primer (5 μM), 0.4 μL of FastPfu Polymerase, and 10 ng of template DNA. PCR products were recovered using 2% agarose gel and purified using the AxyPrep DNA Gel Extraction Kit (Axygen Biosciences, Union City, CA, USA); then, it was eluted by Tris-HCl and was detected by 2% agarose electrophoresis using Quantifluor-ST (Promega, Madison, WI, USA) and quantified according to Illumina Miseq platform (Illumina, San Diego, CA, USA).

### 2.7. Quality Control and Species Annotation

Demultiplexed sequences from each sample were quality filtered and trimmed, de-noised, merged, and then the chimeric sequences were identified and removed using the QIIME2 dada2 plugin to obtain the feature table of amplicon sequence variant (ASV) [22]. The QIIME2 feature-classifier plugin was then used to align ASV sequences to a pre-trained GREENGENES 13_8 99% database (trimmed to the V3V4 region bound by the 338F/806R primer pair) to generate the taxonomy table [23]. Any contaminating mitochondrial and chloroplast sequences were filtered using the QIIME2 feature-table plugin.

### 2.8. Variance Analysis

Appropriate methods, including ANCOM, ANOVA, Kruskal–Wallis, LEfSe, and DEseq2, were employed to identify the bacteria with different abundance among samples and groups.

### 2.9. Diversity Analysis

Diversity metrics were calculated using the core-diversity plugin within QIIME2. Feature level alpha diversity indices, such as observed OTUs (Operational Taxonomic Units), the Chao1 richness estimator, Shannon diversity index, and Faith’s phylogenetic diversity index, were employed to assess the microbial diversity present within each individual sample. Beta diversity distance measurements, including Bray–Curtis, unweighted UniFrac, and weighted UniFrac, were performed to investigate the structural variation of microbial communities across samples and then visualized via principal coordinate analysis (PCoA).

### 2.10. Association Analysis

Co-occurrence analysis was performed by calculating Spearman’s rank correlations between predominant taxa, and the network plot was used to display the associations among taxa.

### 2.11. Function Analysis

Furthermore, the potential functional profiles of microbial communities were predicted utilizing PICRUSt (Phylogenetic Investigation of Communities by Reconstruction of Unobserved States). The KEGG (Kyoto Encyclopedia of Genes and Genomes) functional annotation was employed to correlate microbial genes with biological pathways, such as metabolism and biosynthesis, through the KEGG database. This analytical approach revealed enriched metabolic functions within the microbiome derived from metagenomic or 16S rRNA data. By aligning 16S rRNA sequences with reference genomes from databases such as Greengenes and SILVA, we were able to infer KEGG pathways and enzyme abundances. This methodology facilitates the generation of hypotheses regarding microbiome/host interactions without the necessity of shotgun sequencing.

### 2.12. Statistical Analysis

All data were analyzed using the Statistical Package for the Socialences, SPSS 26 (IBM Corp, Chicago, IL, USA), and GraphPad Prism (GraphPad Software 9.0, Inc., San Diego, CA, USA). The results were provided with mean and standard deviation (SD), and significant differences were calculated by Duncan’s multiple range test, with *p*-values of 0.05 considered significant and a *p*-value of 0.01 considered exceptionally significant.

## 3. Result

### 3.1. The Differential Contents of Daidzein, Glycitein, and Genistein in SSPs

**Figure 2:**
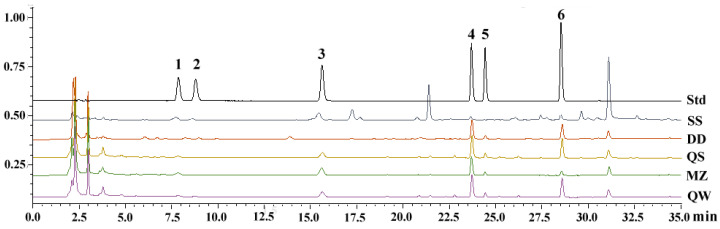
HPLC chromatogram of mixed content in standards and sample. (1)—Daidzin, (2)—Glycitin, (3)—Genistin, (4)—Daidzein, (5)—Glycitein, (6)—Genistein.

**Figure 3:**
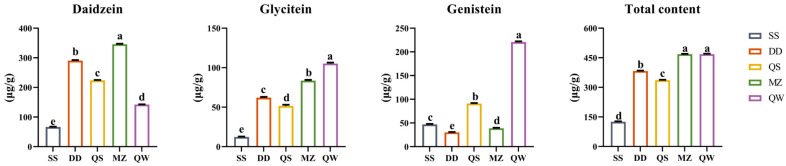
Determination results of aglycones in different fermentation processes (X ± S). Values are expressed as mean ± SD (*n* = 3). Means in the same column with different letters are significantly different by Duncan’s multiple range test (*p* < 0.05). SS—raw soybeans, DD—the fermented group without Chinese medicine, QS—the fermented group of Artemisia Annuae Herba and Mori Folium, MZ—the fermented group of Ephedrae Herba and Perillae Folium, QW—the fermented group of qingwenjiedu decoction.

The β-glucosidase enzyme produced during soybean fermentation hydrolyzes isoflavone glycosides such as genistein, daidzin, and glycitin into isoflavone aglycones like genistein, daidzein, and glycitein. To detect changes in aglycone content during different fermentation processes, HPLC was used (Figure 2). As shown in Figure 3, daidzein, glycitein, and genistein content significantly increased in SSPs compared to raw soybeans. The order of daidzein content in SSPs prepared by different processes was MZ > DD > QS > QW, while the order of glycitein and genistein content was QW > MZ > DD > QS and QW > QS > MZ > DD, respectively. The total aglycones were highest in MZ and QW, and the content of the three isoflavone glycosides varied significantly among different fermentation processes, with MZ having the highest daidzein content and QW having the highest glycitein and genistein content.

### 3.2. The Differential Contents of Amino Acid in SSPs

The comprehensive analysis of the 16 free amino acids and γ-aminobutyric acid (GABA) within the four SSP samples underwent statistical examination, as delineated in Table 1. The cumulative amino acid concentrations in SSPs derived from different fermentation processes exhibited the following: QW > MZ > QS > DD > SS, with a notably significant distinction observed between QW and DD. Furthermore, the assay identified eight essential amino acids in the four SSPs, with distinct concentration profiles: Threonine (Thr), Tryptophan (Trp): MZ > QS > DD > QW; Isoleucine (Ile), Valine (Val), Leucine (Leu): QW > MZ > QS > DD, Methionine (Met): QW > DD > MZ > QS, Phenylalanine (Phe): QW > MZ > DD > QS, and Lysine (Lys): MZ > QS > QW > DD. The divergent patterns of the remaining nine non-essential amino acids are delineated in the table. Of note is the concurrent elevation of the neurotransmitters glutamate and GABA following fermentation. However, GABA, as an inhibitory neurotransmitter, exhibited no significant variance between the four fermentation processes, while the excitatory neurotransmitter glutamate demonstrated a significantly higher concentration in the QW group compared to the DD group.

### 3.3. Dynamic Changes in the Diversity and Profile of the Gut Microbiota over Time

**Figure 4:**
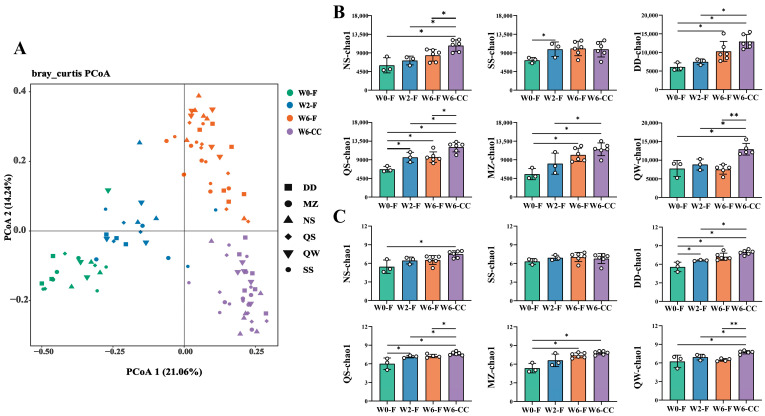
Dynamic changes in the diversity and profile of the gut microbiota in the different fermentation processes. Bray–Curtis’s distance based on the species’ relative abundance was calculated (R“vagan”). (**A**) The α diversity was measured from; (**B**) Chao 1 and (**C**) Shannon. Means in the same column with different pointers are significantly different from Kruskal–Wallis test. Statistic labels: * *p* < 0.05, ** *p* < 0.01. W0-F—fecal samples collected prior to supplementing, W2-F—fecal samples obtained after two weeks of supplementing, W6-F—fecal samples acquired, W6-CC—intestinal contents analyzed after six weeks of supplementing.

In this study, we collected 72 fecal samples at three time points (W0-F, W2-F, W6-F) and 36 colon content samples (W6-CC) from six groups of rats. The Bray–Curtis distance, which is based on the count statistics of OTUs, was used to compare the differences in the composition of microorganisms in the two communities. Principal coordinate analysis (PCoA) was used to simplify the data structure by decomposing the sample distance matrix and to show the natural distribution of the samples at a particular distance scale. Based on the Bray–Curtis distance at a species level using R 4.1.3 software, we conducted PCoA to conclude the differences in the gut microbiota of the six groups at three time points. In the PCoA, the gut microbiota of each experimental group showed similar changes in different growth states (Figure 4A). In addition, the α diversity of fecal and colon content at different time points further demonstrated the dynamic changes in the profile of gut microbiota (Figure 4B,C, Tables S1–S3). The results showed that, except for the QW group, the diversity of the gut microbiota of rats increased progressively with age. Rats consuming SS and QS showed markedly enhanced Chao1 at week 2 compared to rats at week 0. Meanwhile, the Chao1 index was significantly higher in DD, QS, and MZ rats at week 6 compared with week 0 (Figure 4B). Compared with week 0, the Shannon indices of MZ and DD groups increased significantly at week 6, but the Shannon index of QS and QW had no significant difference. Specifically, there was no significant difference between NS and SS in the gut microbiota of separate weeks.

### 3.4. Gut Microbiota Shifts Induced by Fermented and Unfermented Soybeans in Rats

**Figure 5:**
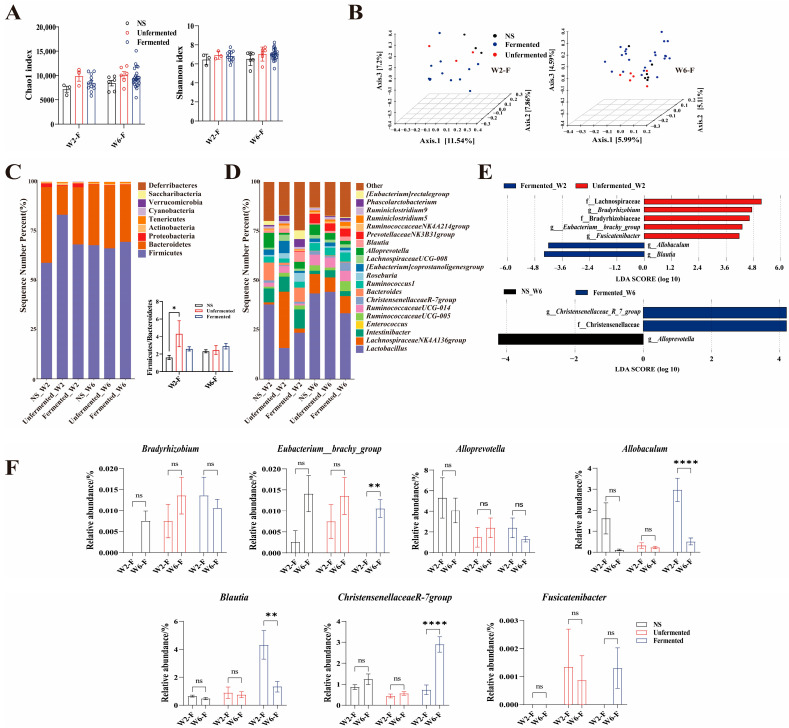
Major shifts of gut bacteria in rats consuming SSP and unfermented soybean. (**A**). The alpha diversity of Chao 1 and Shannon. (**B**). unweighted_unifrac PCoA, Fermented_W2 vs. Unfermented_W2: *p* = 0.041, Fermented_W6 vs. Unfermented_W6: *p* = 0.019 (**C**). Phylum and F/B ratio (**D**). Genus (**E**). LEfSe in genus level (LDA > 4) (**F**). Dynamic changes of genus by LEfSe over time. Statistic labels: ns, not significant, * *p* < 0.05, ** *p* < 0.01, **** *p* < 0.0001.

In this experiment, fermented soybeans included SSPs that were produced by four natural fermentation processes. To investigate the potential beneficial effects of SSPs on the gut microbiota compared to unfermented soybeans, we analyzed the gut microbiota of both groups of rats at week 2 and week 6. As shown in Figure 5A, the α-diversity (Shannon and Chao1) indicated that the distribution of gut microbiota diversity was similar in different groups of rats and did not change significantly over time. However, in the β-diversity analysis, principal coordinate analysis (PCoA) results demonstrated the presence of two distinct clusters at week 6 and week 2 (Figure 5B). These findings suggest that the community profile and composition of the gut microbiota in rats consuming SSPs gradually differ significantly from those consuming unfermented soybeans over time. Based on the diversity analysis results, the phylum and genus levels of gut bacteria were studied for each group. As shown in Figure 5C, the relative abundances of the top 10 phyla indicated that the proportions of firmicutes and bacteroidetes (F/B) were notably greater in the unfermented group compared to the control group at week 2 (*p* < 0.01).

In contrast, the ratios of firmicutes-to-bacteroidetes (F/B) were not significantly different among the three groups at week 6. At the genus level (Figure 5D), the relative abundance of *Lactobacillus* in each group increased with increasing consumption cycles, especially in the unfermented soybean group. To illustrate the microbiota with significant differences between groups at week 2 and week 6, histograms of the LDA values were used. The LEfSe analysis revealed dynamic changes in the relative abundance of microbial communities between the fermentation and unfermented groups at week 2 and week 6. At week 2, the unfermented group exhibited significant upregulation of *Bradyhizobium*, *Eubacterium_brachy_group*, and *Fusicatenibacter*, while the SSPs showed upregulation of *Allobaculum* and *Blautia*. At week 6, *Christensenellaceae_R_7_group* was the differential genus for the fermented_W6 group, while *Alloprevotella* was the differential genus for the NS group (Figure 5E). Furthermore, we observed significant changes over time at the genus level in *Allobaculum*, *Blautia*, *Eubacterium_brachy_group*, and *Christensenellaceae_R_7_group* of the SSP (Figure 5F).

### 3.5. Extended SSPs Ingestion Boosts Gut Microbiota Differences

**Figure 6:**
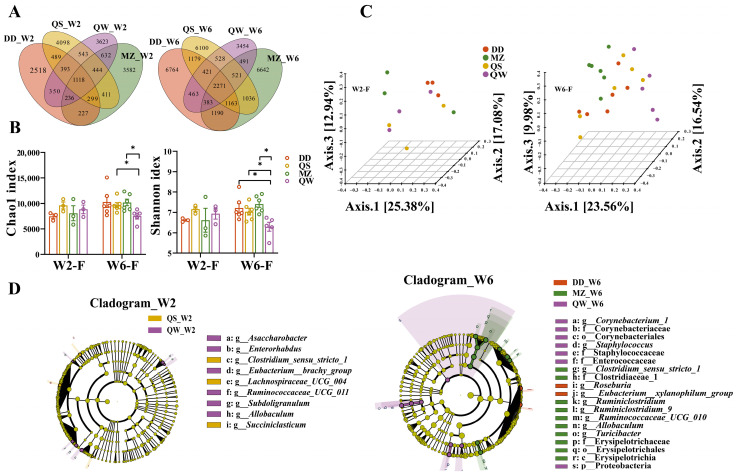
Major shifts of gut bacteria in rats consuming fermented and unfermented soybeans. (**A**). Venn (**B**). The alpha diversity of Chao 1 and Shannon. (**C**). unweighted_unifrac PCoA DD_W6 vs. MZ_W6: *p* = 0.038, DD_W6 vs. QW_W6: *p* = 0.012, MZ_W6 vs. QW_W6: *p* = 0.044, QS_W6 vs. QW_W6: *p* = 0.016. (**D**). LEfSe in genus level (LDA > 3). Statistic labels: ns, not significant, * *p* < 0.05.

The fermented soybeans in this study contained four types of SSPs with different fermentation processes. Venn diagrams were employed to visually demonstrate the commonalities in abundance across the groups, highlighting the shared characteristics between them. The Venn diagrams illustrated an overall increase in OTUs across most experimental groups and the occurrence of shared OTUs between groups heightened at week 6. Conversely, a reduction in OTUs was observed in the QW group, accompanied by a noteworthy decrease in shared OTUs with QS and MZ (Figure 6A). Following this, the impact of the four SSPs on the α- diversity of the gut microbiota was evaluated using the Chao 1 and Shannon diversity indices. At week 2, no significant difference in α-diversity and β-diversity was observed, indicating that the consumption of the four SSPs did not significantly impact the regulation of gut microbiota diversity. However, by week 6, the Chao1 and Shannon diversity indices were found to be lower in the QW group compared to the MZ and QS groups. This suggests that the inclusion of qingwenjiedu decoction and Artemisiae Annuae Herba as an excipient in the fermentation of SSP may inhibit the richness and diversity of the gut microbiota in rats (Figure 6B). PCoA analysis of genus-level revealed a significant separation in the profile of the gut microbiota among the four groups at week 6 (Figure 6C). Subsequent analysis of the genus-level composition of the gut microbiota in the four SSP groups unveiled an augmentation in *Lactobacillus*, *Lachnospiraceae_NK4A136_group*, *Ruminococcaceae_UCG_014*, *Ruminococcus_1*, and *Ruminococcaceae_UCG_005* following at week 6. Conversely, there was a reduction in *Intestinibacter*, *Bacteroides*, and *Blautia* (Figure S1A).

Discriminative features of bacterial taxa were identified with an LDA score >3.0. After SSP consumption for two weeks, specific genera were exclusively present in the QW and QS groups. The relative abundance of gut microbiota in the QS_W2 group demonstrated a significant up-regulation in *Clostridium_sensu_stricto_1*, *Succiniclasticum*, *Lachnospiraceae_UCG_004*, and *Ruminococcaceae_UCG_011*. Similarly, in group QW_W2, the relative abundance of genera *Allobaculum*, *Enterorhabdus*, *Eubacterium_brachy_group*, *Subdoligranulum*, and *Asaccharobacter* exhibited a significant up-regulation. However, after six weeks of continuous SSP consumption, specific genera were observed in the QW, DD, and MZ groups, indicating that the modulation of the gut microbiota by the four SSP species was influenced by the duration of consumption (Figure 6D). The QW_W6 group was dominated by the phylum Proteobacteria, order Corynebacteriales, and families Corynebacteriaceae, Staphylococcaceae, and Enterococcaceae, while the rats consuming MZ_W6 showed the highest relative abundance of the classes Erysipelotrichia and families Erysipelotrichaceae and Clostridiaceae_1. At the genus level, DD_W6 had the highest relative abundance of the genera *Roseburia* and *Eubacterium_xylanophilum_group*, whereas QW_W6 and MZ_W6 had the genera *Corynebacterium_1* and *Staphylococcus*, and *Allobaculum*, *Turicibacter*, *Ruminiclostridium_9*, *Clostridium_sensu_stricto_1*, *Ruminococcaceae_UCG_010*, and *Ruminiclostridium*, all significantly up-regulated. Refer to Table 2 for detailed information.

### 3.6. SSPs’ Impact on Neurotransmitters and the Correlation Between Gut Microbiota, Neurotransmitters, and Isoflavones

**Figure 7:**
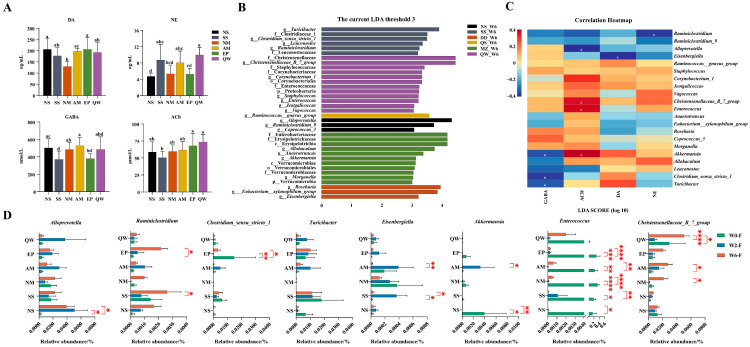
Correlation analysis of altered microbial genus affected by ACH, DA, GABA, NE (**A**). The ACH, DA, GABA, and NE contents in serum. (**B**). LEfSe in genus level (LDA > 3). (**C**). Pearson correlation between microbial and the neurotransmitter (negative values represent negative correlation, while positive values represent positive correlation) (**D**). The dynamics of specific genera over time in each group. Different letters are significantly different. Statistic labels: ns, not significant, * *p* < 0.05, ** *p* < 0.01, *** *p* < 0.001, **** *p* < 0.0001.

Neurotransmitters were evaluated in the serum of rats consuming SSPs and raw soybeans for six weeks. The results showed that each of the four SSPs had a distinctive modulation of neurotransmitters. In Figure 7A, DA—dopamine levels—exhibited a decrease in all four SSP groups compared to the NS group, with a significant down-regulation observed in the DD group. The DA levels in the DD group were markedly lower than those in the QS and MZ groups. NE—norepinephrine levels—showed an increase in all four SSP groups compared to the NS group, with a significant elevation observed in the QW and QS groups. NE was significantly higher in the QW group than in the DD and MZ groups. GABA-γ-aminobutyric acid levels in the MZ group were significantly lower than those in the NS and QS groups. ACH—acetylcholine content—exhibited an elevation in all four SSP groups, although without significant differences.

To investigate whether changes in the abundance of the gut microbiota within each group were correlated with the presence of neurotransmitters, we conducted further analysis on the correlation between specific strains and neurotransmitters (LDA > 3), as shown in Figure 7B. The findings suggest significant and negative correlations between *Ruminiclostridium*, *Eisenbergiella*, and *Alloprevotella* with NE, DA, and ACH levels, respectively. GABA exhibited a negative correlation with three genera: *Akkermansia*, *Clostridium_sensu_stricto_1*, and *Turicibacter*. In contrast, *Enterococcus* and *Christensenellaceae_R_7_group* showed significant and positive correlations with ACH (Figure 7C).

Considering the correlation results, a subsequent analysis was conducted to examine the temporal changes in genera exhibiting significant correlations following the consumption of various types of SSPs by rats (Figure 7D). The abundance of *Ruminiclostridium* and *Christensenellaceae_R_7_group* increased after consuming for six weeks, with a significant increase in *Ruminiclostridium* in the MZ group and a significant increase in *Christensenellaceae_R_7_group* in QS and QW. The abundance of *Clostridium_sensu_stricto_1* and *Enterococcus* decreased in abundance after consuming for six weeks, with *Ruminiclostridium* showing a significant decrease in the MZ group and Enterococcus in QS, MZ, and QW. The abundance of *Alloprevotella* and *Turicibacter* increased, while *Eisenbergiella* and *Akkermansia* decreased in the SSPs group; the groups did not change significantly over time. Subsequently, we conducted a further correlation analysis between the content of isoflavones and the genera associated with neurotransmitters. The analysis revealed a positive correlation between *Akkermansia* and daidzein, a negative correlation between *Clostridium_sensu_stricto_1* and glycitein, and a positive correlation between *Enterococcus* and genistein, *Christensenellaceae_R_7_group* was positively correlated with glycitein and genistein (Figure S2).

### 3.7. Bacterial Functions

**Figure 8:**
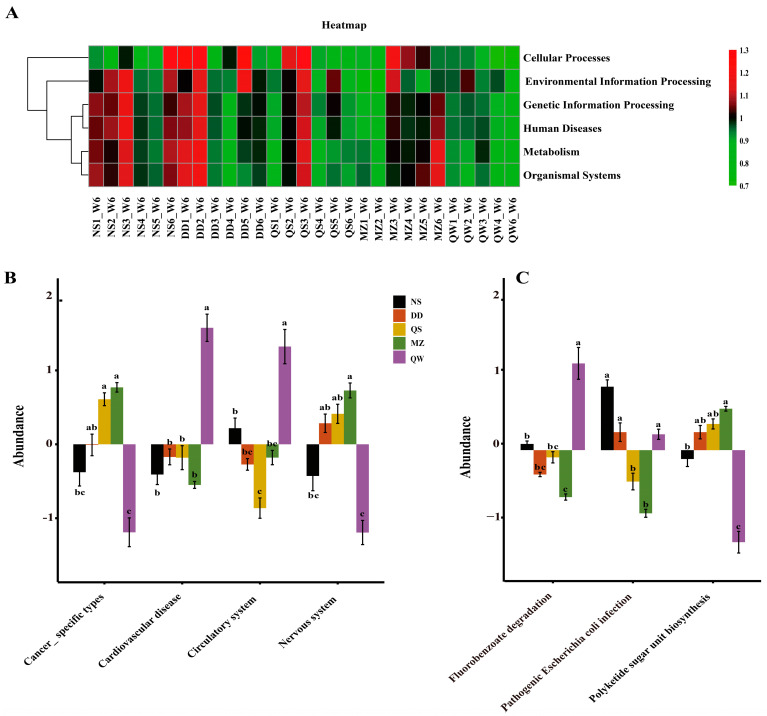
Bacterial gene function annotation and phenotype prediction. (**A**). The heatmap of KEGG functional pathways at the firstly classification level of gut microbiota; (**B**). Result of gut microbiota function prediction at the secondary classification level (different letters are significantly different); (**C**). Result of gut microbiota function prediction at the third classification level (different letters are significantly different).

Furthermore, we predicted the function of the microbiota and found that the metabolic pathway activities of each group are varied (Figure 8A). The results of the secondary function prediction showed that there were significant differences between the QS and MZ groups and the NS group in terms of cancer (type-specific) regulatory function, and significant differences between the QW and QS, MZ, and DD groups. In cardiovascular disease function, the QW group was significantly different from NS and the other three SSPs. In the circulatory system, abundance was significantly down-regulated in QS and significantly up-regulated in QW, and the QW group was significantly different from the other SSPs. In the neurological system, significant differences were found between the MZ, NS, and QW (Figure 8B). Tertiary functional predictions indicated that the degradation of fluorobenzoate was significantly more abundant in the QW group compared to the other groups. Regarding pathogenic *Escherichia coli* infection, the abundance observed in each fermentation group was lower than that in the normal saline group, with the MZ and AM groups exhibiting the lowest levels, which were statistically significant. In the context of polyketide sugar-unit biosynthesis, an increase in abundance was noted for the MZ group, while a decrease was observed for the QW group, with the differences being statistically significant when compared to the other fermentation groups (Figure 8C).

## 4. Discussion

Multilevel analyses in this study indicate that the beneficial regulatory effects on the composition of the normal rat gut microbiota provide robust support for SSPs being considered functional food. At the same time, variations and similarities were observed between SSPs derived from different fermentation processes concerning their effects on neurotransmitters and the regulation of gut microbiota.

The primary constituents of soybeans include isoflavones, peptides, and amino acids. The findings of this experiment indicated variability in the concentrations of isoflavones and amino acids across various preparation methods. Such discrepancies may be attributed to the variations in microbial species and the production of β-glucosidase and protease enzymes [24], which are influenced by the distinct herbs introduced during the fermentation process.

The gut microbiota across various rat populations exhibited a consistent temporal pattern, which can be attributed to rhythmic physiological changes in the animals [25]. Furthermore, the SSPs were found to enhance the diversity of the gut microbiota. The stable firmicutes/bacteroidetes (F/B) ratio observed in SSP-fed rats highlights its potential to mitigate diet-induced dysbiosis, a critical factor in metabolic and inflammatory disorders [26,27,28]. Deviations from this stable ratio, whether elevated or decreased, are indicative of ecological dysbiosis. Unfermented soybeans resulted in an increase in the firmicutes/bacteroidetes (F/B) ratio over a six-week period, whereas fermented soybeans maintained a stable F/B ratio. Additionally, fermented soybeans have been shown to enhance gut microbial diversity and promote a balanced gut microbiome.

In week 2, *Bradyrhizobium* was observed in the specific general consuming unfermented soybeans. In the plant kingdom, some strains of *Bradyrhizobium* form a symbiotic relationship with plant roots, providing a necessary nitrogen source to promote plant growth. However, the role of *Bradyrhizobium* in animals is not well understood. It is possible that these bacteria were detected in the gut of rats after consuming unfermented soy. It has also been reported that some microbiota may be shared between plants and animals, but the underlying mechanism requires further investigation [29]. *Eubacterium_brachy_group* is positively correlated with the secretion of isovaleric, hexanoic, and acetic acids [30]. We observed higher levels of this bacterium in week 2 following the consumption of unfermented soybeans. However, with time, although the abundance of *Eubacterium_brachy_group* increased gradually in all groups, a statistically significant increase was observed only in the fermentation group. Notably, SSP-enriched SCFA-producing taxa (e.g., *Christensenellaceae_R_7_group* and *Eubacterium_brachy_group*), which aligns with emerging evidence that microbial acetate and butyrate enhance intestinal barrier integrity and modulate systemic immunity via epigenetic mechanisms [31,32,33]. At week 2, the fermented group exhibited specific genera, namely *Allobaculum* and *Blautia.* Acetates, propionates, and lactates were identified as crucial fermented metabolites derived from the 13C-labeled glucose by *Allobaculum* [34]. *Blautia* is known for its probiotic properties in transforming and regulating host health and alleviating metabolic syndrome [35]. The abundance exhibited a significant reduction at week 6, yet it persisted in SSPs at levels higher than those observed in the unfermented and normal groups. These findings provide further support for the notion that the consumption of fermented soy products exerts a favorable influence on the gut microbiota. This effect may be attributed to the restoration of metabolic homeostasis through the promotion of bacteria associated with the production of short-chain fatty acids or the amelioration of specific aberrant metabolic processes within the body. Nonetheless, the validation of this hypothesis necessitates the assessment of short-chain fatty acid concentrations.

Significant differences in gut microbiota diversity were observed after consuming SSPs fermented with different processes for six weeks. Bacteria classified within the QS group have been shown to influence gut microbiota associated with metabolic disorders [36,37,38], whereas those in the QW group are involved in the regulation of microbiota related to obesity, with a particular emphasis on *Acharobacter. Acharobacter* is capable of metabolizing soy isoflavones into estragole, which is a significant metabolite exhibiting both estrogenic and antioxidant properties [39]. Furthermore, the predominant genus within the MZ group is instrumental in the regulation of intestinal functions associated with various symptoms and conditions, including obesity [40], inflammation [41,42], and impaired cognitive abilities [43]. SSPs prepared using different processes may be able to improve the gut microecological imbalance related to diseases by regulating the gut microbiota, and there are differences among different processes. These differences may be reflected in aspects such as the types, quantities, and metabolites of the regulated microbiota. This hypothesis still needs to be verified through metabolomics.

During soybean fermentation, various herbs known for their ability to produce bioactive compounds with distinct properties, including the manipulation of microbial growth, were incorporated as excipients [44,45,46]. Previous studies have shown that the incorporation of herbs as excipients when preparing SSPs can modulate the microbial composition of the koji, promoting the growth of beneficial microorganisms while inhibiting the proliferation of spoilage and pathogenic microorganisms [47]. Variations in microbial species can instigate diverse biotransformation processes. As previously noted, the disparities in isoflavone and amino acid content across different species may be attributed to variations in the types and abundance of microorganisms produced during the fermentation process. Therefore, we posit that the functional active ingredients generated through microbial-regulated transformations during SSP fermentation across various varieties likely exert distinct effects on the gut microbiota, aligning with our anticipated results.

The brain–gut axis is an increasingly studied aspect in the field of neurological disorders. Gut microbiota may influence the central nervous system by producing signaling molecules that are integral to the brain–gut axis. The Chinese Pharmacopoeia recognizes the potential of SSP in alleviating vexation. Dopamine and norepinephrine, both belonging to the catecholamine neurotransmitter family, play crucial roles in regulating motor neurons, spatial memory function, motivation, awakening, reward, and pleasure. Moreover, they are implicated in the prevention and control of various neurodegenerative diseases [48,49]. Acetylcholine, a vital neurotransmitter in the central cholinergic system, is associated with memory and the maintenance of consciousness. In this experiment, DA in the DD group exhibited a significant decrease, while the levels in other groups were comparable to those of the normal group. Correlation analysis revealed a significant negative correlation between DA and *Eisenbergiella*. By studying the dynamics of this bacterium in each rat group, we observed a gradual decrease in its abundance in the DD group, whereas the other groups initially showed an increase followed by a decrease. Unfortunately, the impact of *Eisenbergiella* on neurotransmitters remains unexplored. Genistein in the DD group SSP was lower compared to the other groups. Genistein has been reported to increase DA levels [50]. However, no correlation was observed between genistein and *Eisenbergiella*, indicating that genistein may regulate dopamine levels through alternative pathways. The level of NE in the QS and QW groups was significantly higher than that in the control group. Additionally, the abundance of *Ruminiclostridium*, which exhibited a negative correlation with norepinephrine, did not display a consistent pattern among the groups. Although fermented soybean products increase GABA levels, in this study, the DD, QS, and QW groups showed a non-significant increase, and the MZ group even exhibited a decrease in GABA levels in serum. Constituent and genus correlation analysis showed a positive correlation between daidzein and *Akkermansia.* Among the different fermentation processes, the MZ group exhibited the highest levels of daidzein. We propose that this constituent may regulate the secretion of GABA in the serum by modulating the gut microbiota. ACH did not show substantial changes, and the QW and MZ groups exhibited higher levels compared to the QS group. Although the regulation of ACH by each group was not significant, the specific genus *Christensenellaceae_R_7_group* in the QW group showed a positive correlation with the levels of both genistein and glycitein. Moreover, the QW group exhibited the highest levels of ACH compared to the other groups. These observations suggest a potential link between *Christensenellaceae_R_7_group* and ACH regulation. These findings indicate that SSPs from different fermentation processes may induce differential regulation of gut microbiota, thereby stimulating varied neurotransmitter secretion. Alternatively, different functional components may directly influence the secretion of neurotransmitters.

## 5. Conclusions

This study is the first to analyze the efficacy of various fermentation processes of four types of SSPs with traditional Chinese characteristics in enhancing the abundance of beneficial bacteria and regulating neurotransmitters, attributed to the addition of diverse excipients. Furthermore, the experiment suggests that distinct fermentation processes of SSPs can selectively regulate various neurotransmitters by integrating the modern theory of the gut–brain axis with the characteristic of “relieving depression and removing anxiety”. We propose that bioactive compounds from SSPs regulate gut microbiota, subsequently stimulating neurotransmitter production, or that different functional components have direct effects on neurotransmitter secretion. The details need to be further explored. These results can be attributed to (i) the presence of diverse microbial species resulting from the addition of various herbal excipients and (ii) the distinct enzymes produced through microbial metabolism. Our findings highlight that SSPs, as traditional functional foods, uniquely regulate gut microbiota and neurotransmitter levels. The current investigation into the neurological modulation of the SSPs was limited to the examination of select neurotransmitters and did not encompass more extensive assessments, including behavioral and cognitive evaluations, nor did it involve thorough explorations of the brain–gut axis mechanisms. Future research should be structured to elucidate the distinctions in neurological regulation and the brain–gut axis across the four SSPs, thereby establishing a robust foundation for their utilization in health-related applications.

## Figures and Tables

**Table 1:** Composition and content of free amino acids in different fermentation processes.

Free Amino Acid	Free Amino Acids Content (mg/g)				
	SS	DD	QS	MZ	QW
Aspartic Acid (Asp)	0.43 ± 0.07 ^c^	2.12 ± 0.06 ^bc^	2.43 ± 0.28 ^ab^	2.81 ± 0.49 ^ab^	3.70 ± 0.53 ^a^
Glutamic Acid (Glu)	0.65 ± 0.11 ^c^	4.21 ± 0.12 ^bc^	4.48 ± 0.55 ^abc^	5.05 ± 0.92 ^ab^	6.60 ± 0.94 ^a^
Serine (Ser)	0.23 ± 0.05 ^b^	0.19 ± 0.01 ^b^	1.82 ± 0.23 ^a^	2.06 ± 0.35 ^a^	0.34 ± 0.05 ^ab^
Glycine (Gly)	0.26 ± 0.05 ^d^	1.71 ± 0.05 ^b^	1.17 ± 0.14 ^c^	1.32 ± 0.20 ^c^	2.22 ± 0.25 ^a^
Threonine (Thr)	0.38 ± 0.12 ^c^	0.96 ± 0.03 ^bc^	1.55 ± 0.23 ^ab^	1.82 ± 0.38 ^a^	0.84 ± 0.87 ^c^
Proline (Pro)	0.21 ± 0.07 ^c^	0.67 ± 0.06 ^b^	1.31 ± 0.34 ^a^	1.30 ± 0.40 ^a^	1.17 ± 0.25 ^a^
Alanine (Ala)	0.61 ± 0.12 ^c^	2.46 ± 0.09 ^bc^	2.41 ± 0.30 ^bc^	2.60 ± 0.45 ^ab^	3.59 ± 0.48 ^a^
γ-Aminobutyric Acid (GABA)	0.00 ± 0.00 ^b^	0.43 ± 0.03 ^ab^	0.60 ± 0.01 ^a^	0.60 ± 0.14 ^a^	0.60 ± 0.10 ^a^
Tryptophan (Trp) *	0.25 ± 0.05 ^d^	0.72 ± 0.05 ^b^	0.56 ± 0.09 ^c^	0.66 ± 0.14 ^bc^	0.97 ± 0.13 ^a^
Methionine (Met) *	0.12 ± 0.04 ^d^	0.89 ± 0.05 ^b^	0.55 ± 0.09 ^c^	0.75 ± 0.16 ^b^	1.35 ± 0.20 ^a^
Valine (Val) *	0.33 ± 0.08 ^c^	2.13 ± 0.09 ^bc^	2.20 ± 0.31 ^ab^	2.42 ± 0.48 ^ab^	3.29 ± 0.47 ^a^
Phenylalanine (Phe) *	0.45 ± 0.12 ^c^	2.63 ± 0.14 ^bc^	2.51 ± 0.41 ^bc^	2.85 ± 0.59 ^ab^	3.87 ± 0.53 ^a^
Isoleucine (Ile) *	0.26 ± 0.07 ^c^	1.95 ± 0.08 ^bc^	2.13 ± 0.32 ^ab^	2.32 ± 0.46 ^ab^	3.02 ± 0.42 ^a^
Leucine (Leu) *	0.55 ± 0.18 ^c^	3.82 ± 0.15 ^b^	4.00 ± 0.61 ^b^	4.41 ± 0.85 ^b^	7.19 ± 0.97 ^a^
Lysine (Lys) *	0.69 ± 0.16 ^c^	1.79 ± 0.15 ^b^	3.04 ± 0.60 ^a^	3.10 ± 0.77 ^a^	2.89 ± 0.45 ^a^
Histidine (His) *	0.23 ± 0.04 ^b^	0.46 ± 0.04 ^a^	0.16 ± 0.11 ^b^	0.18 ± 0.19 ^b^	0.75 ± 0.13 ^a^
Arginine (Arg)	2.26 ± 0.51 ^a^	0.33 ± 0.04 ^c^	2.81 ± 0.57 ^a^	2.85 ± 0.77 ^ab^	0.74 ± 0.14 ^b^
Total	7.91 ± 1.74 ^c^	27.48 ± 1.19 ^bc^	33.75 ± 5.26 ^ab^	37.11 ± 7.67 ^ab^	43.13 ± 5.93 ^a^

Values are mean ± SD (*n* = 4). * Essential amino acids. Different superscripts within a row meant significant differences at *p* < 0.05.

**Table 2:** The summary from phylum to genus of bacteria taxa with LDA scores of higher than 3 and their clusters.

Group	Kingdom	Phylum	Class	Order	Family	Genus	LDA	*p* Value
QS_W2	Monera	Firmicutes	Clostridia	Clostridiales	Clostridiaceae_1	*Clostridium_sensu_stricto_1*	3.47	0.04763
QS_W2	Monera	Firmicutes	Negativicutes	Selenomonadales	Acidaminococcaceae	*Succiniclasticum*	3.45	0.02542
QS_W2	Monera	Firmicutes	Clostridia	Clostridiales	Lachnospiraceae	*Lachnospiraceae_UCG_004*	3.33	0.03290
QW_W2	Monera	Firmicutes	Erysipelotrichia	Erysipelotrichales	Erysipelotrichaceae	*Allobaculum*	4.46	0.03290
QW_W2	Monera	Actinobacteria	Coriobacteriia	Coriobacteriales	Coriobacteriaceae	*Asaccharobacter*	3.67	0.04987
QW_W2	Monera	Firmicutes	Clostridia	Clostridiales	Family_XIII	*Eubacterium__brachy_group*	3.41	0.02232
QW_W2	Monera	Firmicutes	Clostridia	Clostridiales	Ruminococcaceae	*Subdoligranulum*	3.33	0.03781
QW_W2	Monera	Firmicutes	Clostridia	Clostridiales	Ruminococcaceae	*Ruminococcaceae_UCG_011*	3.22	0.04987
QW_W2	Monera	Actinobacteria	Coriobacteriia	Coriobacteriales	Coriobacteriaceae	*Enterorhabdus*	3.06	0.04148
DD_W6	Monera	Firmicutes	Clostridia	Clostridiales	Lachnospiraceae	*Roseburia*	3.94	0.02590
DD_W6	Monera	Firmicutes	Clostridia	Clostridiales	Lachnospiraceae	*Eubacterium_xylanophilum_group*	3.78	0.00894
MZ_W6	Monera	Firmicutes	Erysipelotrichia	Erysipelotrichales	Erysipelotrichaceae	*Allobaculum*	3.87	0.02810
MZ_W6	Monera	Firmicutes	Erysipelotrichia	Erysipelotrichales	Erysipelotrichaceae	*Turicibacter*	3.87	0.00202
MZ_W6	Monera	Firmicutes	Clostridia	Clostridiales	Ruminococcaceae	*Ruminiclostridium_9*	3.63	0.01729
MZ_W6	Monera	Firmicutes	Clostridia	Clostridiales	Clostridiaceae_1	*Clostridium_sensu_stricto_1*	3.44	0.00431
MZ_W6	Monera	Firmicutes	Clostridia	Clostridiales	Ruminococcaceae	*Ruminococcaceae_UCG_010*	3.29	0.02514

## Data Availability

The original contributions presented in the study are included in the article/Supplementary Material; further inquiries can be directed to the corresponding author.

## Supplementary Materials

The following supporting information can be downloaded at https://www.mdpi.com/article/10.3390/foods14040671/s1. Table S1. Changes of microbial function diversity—faith_pd, Table S2. Changes of microbial function diversity—observed_otus. Table S3. Changes of microbial function diversity—simpson. Figure S1. The composition of gut microbiota in SSP of different fermentation processes at the phylum level. (A). The genra level at week 2. (B). The genra level at week 6. Figure S2. Correlation analysis between *Akkermansia*, *Clostridium_sensu_stricto_1*, *Enterococcus*, *Christensenellaceae_R_7_group*, and isoflavone content. With Pearson correlation coefficients (R^2^), *p*-values, and 95% confidence intervals for the correlation coefficients presented as statistical parameters to comprehensively describe the strength and significance of the correlations.
