# Supplementary material for: Gut Microbiota and Neurotransmitter Regulation: Functional Effects of Four Traditional Chinese Fermented Soybean (Sojae Semen Praeparatum)
Source: Foods. 2025 Feb 16;14(4):671. doi: 10.3390/foods14040671 (PMC11854601; doi:10.3390/foods14040671)
Supplement: Supplementary file 1 [file foods-14-00671-s001.zip › foods-3461049-supplementary.pdf]

**Table S1** Changes of microbial function diversity -faith_pd

| Treats | W0-F | W2-F | W6-F | W6-CC |
|---|---|---|---|---|
| NS | 98.96±27.16$^{ab}$ | 118.11±12.05$^{ab}$ | 132.44±17.06$^{a}$ | 148.50±18.28$^{b}$ |
| SS | 126.68±15.56 | 155.73±25.20 | 158.49±22.11 | 148.54±22.26 |
| DD | 114.93±14.88$^{b}$ | 132.41±8.10$^{b}$ | 170.59±35.27$^{a}$ | 194.57±25.74$^{a}$ |
| QS | 128.20±15.63$^{c}$ | 162.09±9.39$^{b}$ | 165.57±18.96$^{ab}$ | 182.53±11.62$^{a}$ |
| MZ | 107.64±19.13$^{b}$ | 142.23±40.87$^{ab}$ | 169.86±22.21$^{a}$ | 175.11±11.51$^{a}$ |
| QW | 118.96±34.79$^{b}$ | 147.44±17.36$^{ab}$ | 128.24±13.30$^{b}$ | 179.27±16.84$^{a}$ |

**Table S2** Changes of microbial function diversity -observed_otus

| Treats | W0-F | W2-F | W6-F | W6-CC |
|---|---|---|---|---|
| NS | 2229.00±781.01$^{ab}$ | 2607.00±341.29$^{a}$ | 3044.17±473.90$^{a}$ | 3856.00±525.48$^{b}$ |
| SS | 2648.67±391.48 | 3574.67±714.53 | 3517.83±655.04 | 3596.33±692.98 |
| DD | 2188.33±353.66$^{b}$ | 2572.33±124.34$^{b}$ | 3661.83±929.27$^{a}$ | 4621.50±697.96$^{a}$ |
| QS | 2545.00±399.01$^{c}$ | 3357.67±297.35$^{b}$ | 3475.17±485.60$^{b}$ | 4247.67±336.02$^{a}$ |
| MZ | 2126.00±509.50$^{b}$ | 2815.67±1102.73$^{ab}$ | 3616.50±634.71$^{a}$ | 4067.83±353.55$^{a}$ |
| QW | 2623.67±837.52$^{b}$ | 3195.33±489.06$^{b}$ | 2620.40±363.04$^{b}$ | 4413.00±568.11$^{a}$ |

**Table S3** Changes of microbial function diversity -simpson

| Treats | W0-F | W2-F | W6-F | W6-CC |
|---|---|---|---|---|
| NS | 0.88±0.08$^{a}$ | 0.94±0.05$^{ab}$ | 0.94±0.04$^{ab}$ | 0.97±0.01$^{b}$ |
| SS | 0.93±0.03$^{a}$ | 0.96±0.01$^{b}$ | 0.96±0.03$^{ab}$ | 0.95±0.03$^{ab}$ |
| DD | 0.87±0.08$^{c}$ | 0.96±0.00$^{b}$ | 0.96±0.02$^{ab}$ | 0.98±0.01$^{a}$ |
| QS | 0.90±0.10$^{b}$ | 0.97±0.01$^{ab}$ | 0.96±0.02$^{b}$ | 0.98±0.00$^{a}$ |
| MZ | 0.85±0.07$^{b}$ | 0.96±0.02$^{a}$ | 0.98±0.00$^{a}$ | 0.98±0.01$^{a}$ |
| QW | 0.95±0.02$^{ab}$ | 0.97±0.01$^{ab}$ | 0.92±0.04$^{b}$ | 0.97±0.01$^{a}$ |

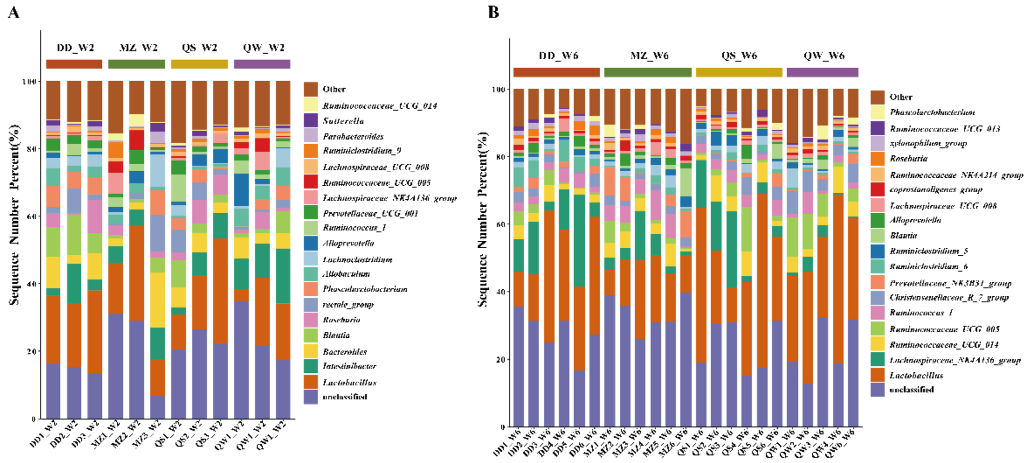


**Figure S1** The composition of gut microbiota in SSP of different fermentation processes at the phylum level. (A). The genra level at week 2. (B). The genra level at week 6

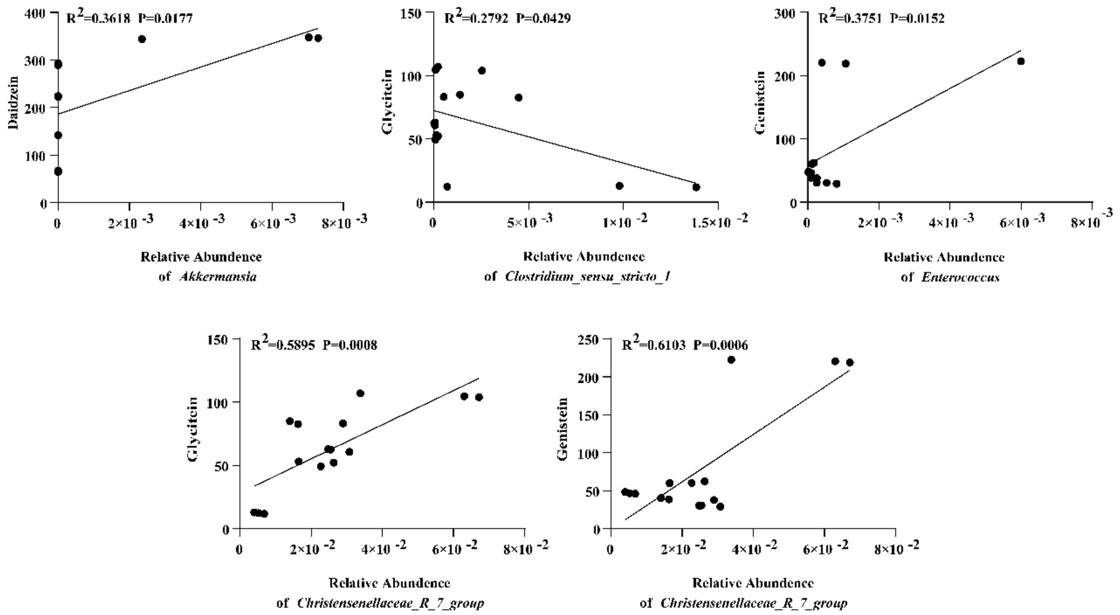


**Figure S2** Correlation analysis between *Akkermansia*, *Clostridium_sensu_stricto_1*, *Enterococcus*, *Christensenellaceae_R_7_group*, and isoflavone content. With Pearson correlation coefficients ($R^2$), P-values, and 95% confidence intervals for the correlation coefficients presented as statistical parameters to comprehensively describe the strength and significance of the correlations.
